# Supplementary material for: Cu(II) Ion Adsorption by Aniline Grafted Chitosan and Its Responsive Fluorescence Properties
Source: Molecules. 2020 Feb 26;25(5):1052. doi: 10.3390/molecules25051052 (PMC7179099; doi:10.3390/molecules25051052)

Supporting Information

Aniline Grafted Chitosan: Adsorption and Fluorescence Behavior Study toward Cu(II) Ions

Authors: Bahareh Vafakish and Lee D. Wilson*
Department of Chemistry, University of Saskatchewan, 110 Science Place, Saskatoon, Saskatchewan, S7N 5C9, Canada
*Correspondence: lee.wilson@usask.ca, Tel.: +1-306-966-2961; Fax: +1-306-966-4730

Fig S1: Proposed chemical structure of CS-Ac-An. R is the acetyl group.

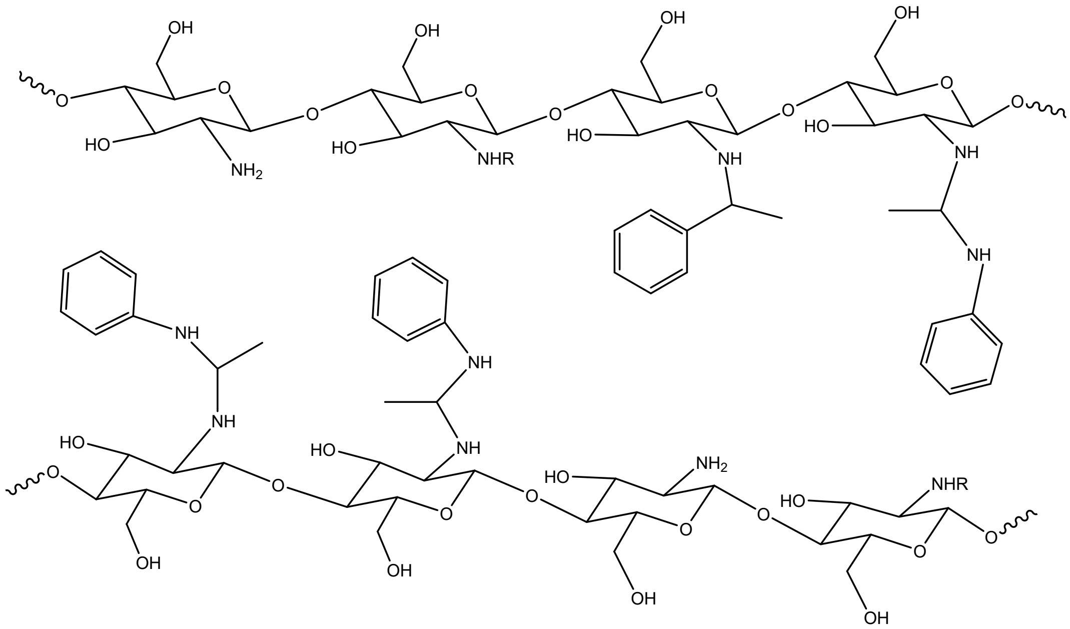

Fig S2: Result of EDX analysis on the flakes after adsorption

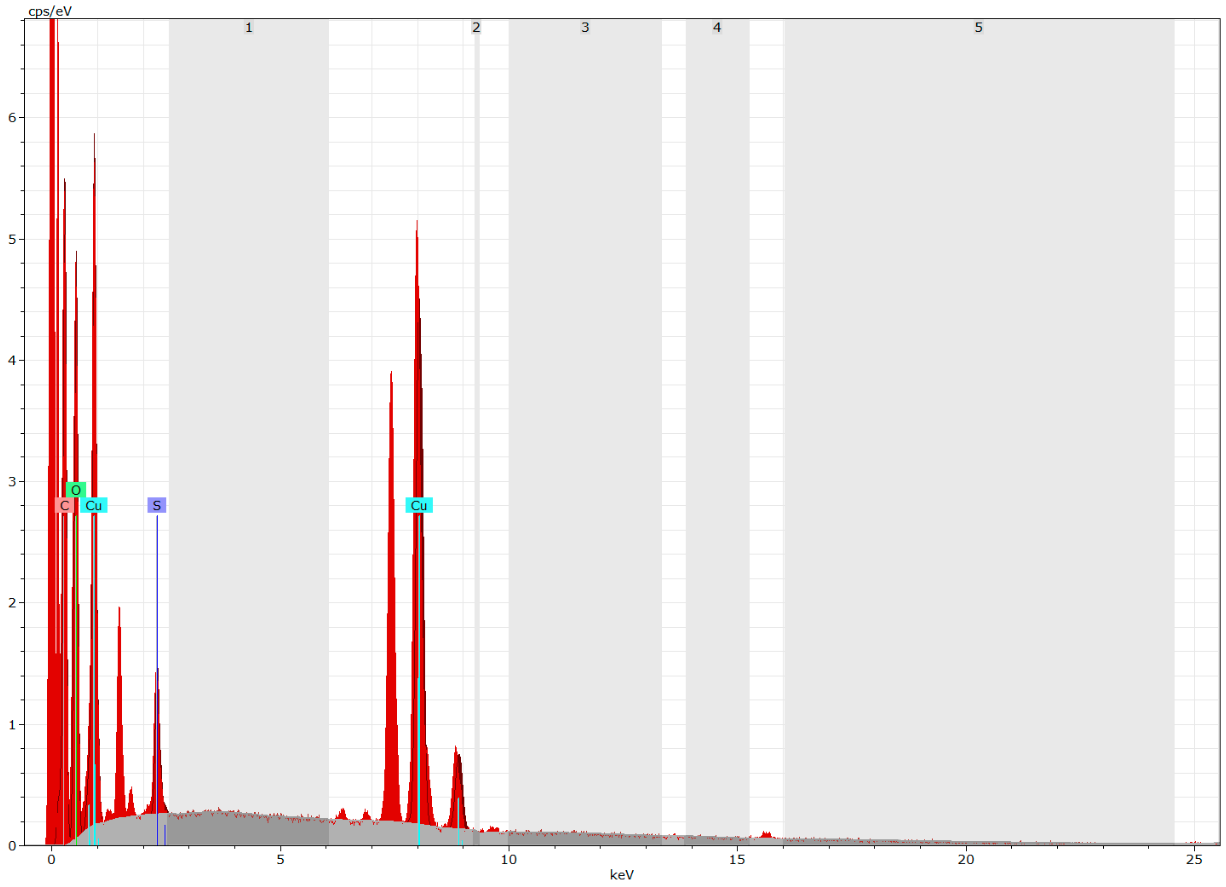

Table S1: Elemental analysis results by the EDX technique

Element	Series	[wt.%]
Carbon	K-series	59.24
Oxygen	K-series	8.154
Sulfur	K-series	4.596
Copper	K-series	17.75
Nitrogen	K-series	10.25

Table S2: Curve fitting results of XPS analysis for C1*s*, N1*s*, O1*s* and Cu 2*p* before and after copper adsorption

Peak	Suggested attribution	BE (eV) before adsorption	BE (eV) after adsorption
C 1*s*	C=C	283.24	283.31
	C-C	284.80	284.83
	C-O, C-N	286.29	286.38
	Cu(II)-π system	-	287.29
N 1*s*	-NH2	396.82	396.85
	-NH3+	397.41	397.42
	-N-Cu(II)	-	399.05
O 1*s*	O-C	529.81	529.87
	O-H	530.48	530.50
Cu 2*p*	$2p_{3/2}$	-	931.02
	Satellite	-	940.04
	$2p_{1/2}$	-	951.02
	Satellite	-	960.04

Figure S3: Proposed structure of adsorbed copper with η=6, sandwiched between two arene ring

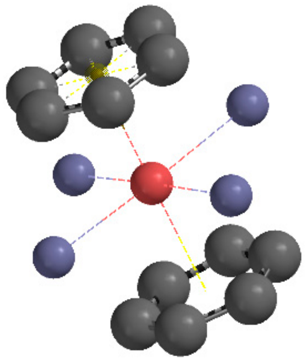

Table S3: Langmuir Fitting Parameters for Cu (II) uptake with CS and CS-Ac-An

**CS**			**CS-Ac-An**		
Q_m (mg g^{-1})	K_L (L g^{-1})	Adj-R^2	Q_m (mg g^{-1})	K_L (L g^{-1})	Adj-R^2
5.7	9.1	0.746	138.1	5.8	0.959

Table S4: Comparison of the adsorption capacity (*Q_m)* of different adsorbents for Cu(II)

Adsorbent	Adsorption capacity (mg g^{-1})	Reference
Polystyrene-supported chitosan	99.8	54
Chitosan in prawn shell	17.1	20
Chitosan-coated sand	8.18	51
Magnetic carboxymethyl chitosan nanoparticles	232	55
Tripolyphosphate crosslinked chitosan beads	15.6	44
Chitosan-derived Schiff bases	32.5	22
Chitosan	4.7	52
Formaldehyde cross-linked modified chitosan–thioglyceraldehyde Schiff's base	76	53
Chitosan-coated mesoporous microspheres of calcium silicate hydrate	425	50
Porous poly(L-lactic acid) (PLLA)-Chitosan	112	56
Aniline grafted chitosan	106.5	This study

Fig S4: (a) Effect of temperature on Cu(II) adsorption from 288 to 308K at ambient pH and temperature. Cu(II) concentration: 100 ppm, contact time: 24 h,(b) Plot of Ln K_e versus 1/T for the determination of ΔH° and ΔS° using the vant' Hoff equation (eq 5)

(a)

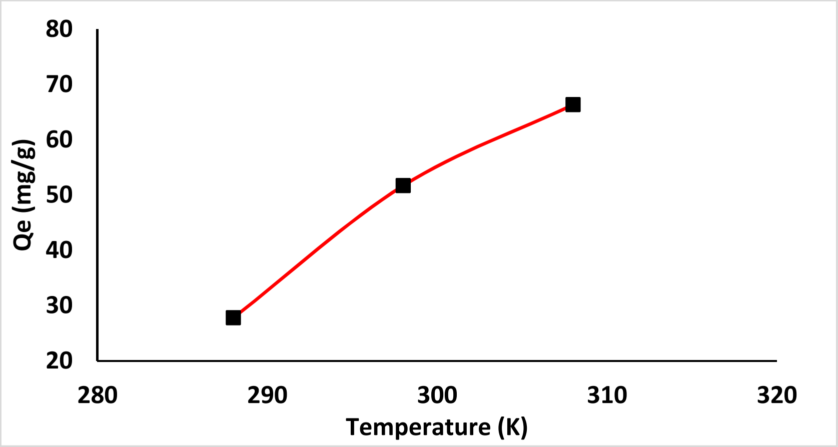

(b)

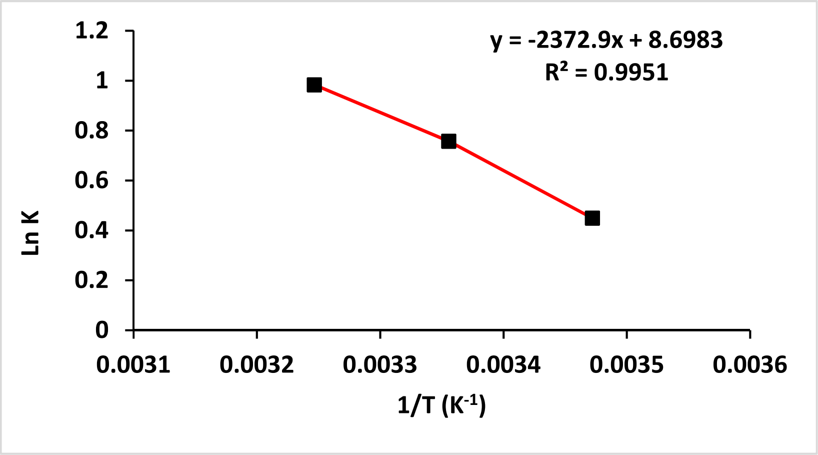

Table S5: Thermodynamic Parameters

Temp (K)	ΔG° (kJ/mol)	ΔH° (kJ/mol)	ΔS° (J/mol.K)	R^2
288	-1.07	19.6	72.2	0.994
298	-1.88			
308	-2.52			

Table S6: PFO and PSO Fitting Parameters

Pseudo First Order (PFO)			Pseudo Second Order (PSO)		
Q_t (mg g^{-1})	k_1 (min^{-1})	R^2	Q_t (mg g^{-1})	k_2 (g mol^{-1} min^{-1})	R^2
28.3	8.56×10^{-3}	0.973	35.3	3.89×10^{-3}	0.982

Table S7: Rate (k_i) values with the unit of (mg/g.min$^{1/2}$)

20 ppm		50 ppm		100 ppm	
k_1	k_2	k_1	k_2	k_1	k_2
1.71	0.303	0.450	0.489	0.142	0.714

Fig S5: Regeneration cycles for CS-Ac-An loaded with Cu(II) . Adsorption cycle condition: copper ion concentration: 100 ppm, contact time: 24 h, adsorbent dosage: 5 mg. Desorption cycle condition: EDTA solution: 0.01 M, contact time 3 h.

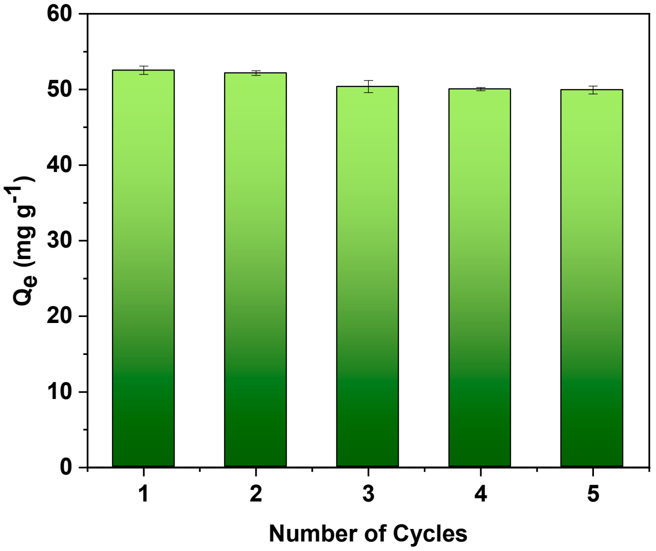

Figure S6: UV-Vis Spectra of CS-Ac-An (1g L^{-1}) in 2% acetic acid from 250-550 nm in the presence of various concentration of Cu(II) ion

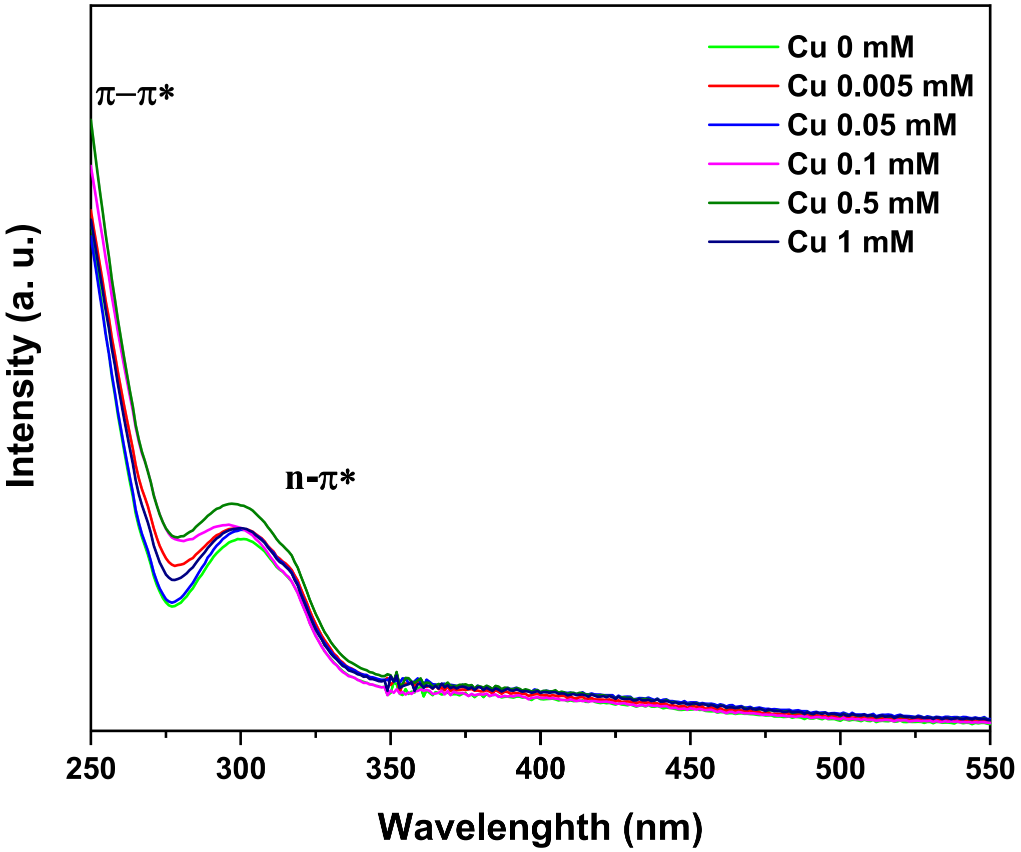

Figure S7: Fluorescence emission spectra of CS-Ac-An at different excitation wavelength

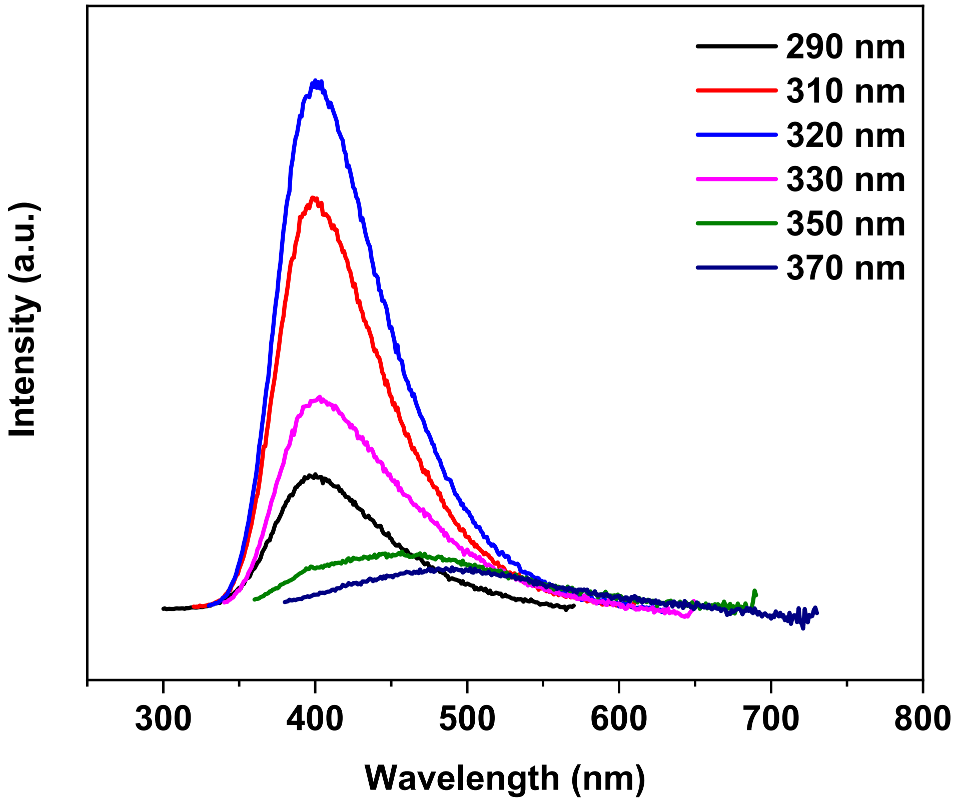

Supplement: Supplementary file 1 [file molecules-25-01052-s001.pdf]
